# Case Report: Supercharged end-to-side anterior interosseous to ulnar motor nerve transfer for ulnar nerve neuropathy after cross pinning of pediatric supracondylar humerus fracture

**DOI:** 10.3389/fped.2024.1398624

**Published:** 2024-09-10

**Authors:** Jian-Jiun Chen, Chen-Yuan Yang

**Affiliations:** ^1^ Department of Orthopedics and Traumatology, Taipei Veterans General Hospital, Taipei, Taiwan; ^2^ Department of Surgery, School of Medicine, National Yang Ming Chiao Tung University, Taipei, Taiwan; ^3^ Department of Orthopedic Surgery, Kuang Tien General Hospital, Taichung, Taiwan; ^4^ Department of Nursing, Hungkuang University, Taichung, Taiwan

**Keywords:** supracondylar distal humerus fracture, ulnar neuropathy, supercharged end-to-side nerve transfer, pediatric, case report

## Abstract

Ulnar nerve neuropathy following pediatric supracondylar humerus fracture fixation with cross pinning poses challenges in management. Despite various treatment strategies, including conservative approaches and early intervention, achieving complete neural recovery remains elusive in some cases. This paper presents a novel approach utilizing supercharged end-to-side anterior interosseous nerve transfer for a 13-year-old patient who experienced persistent ulnar neuropathy after K-wire removal. The patient underwent neurolysis of the ulnar nerve followed by nerve transfer, resulting in significant improvement in function and strength. This case highlights the potential efficacy of combining neurolysis and supercharge techniques in pediatric ulnar neuropathy cases refractory to conservative treatment, offering a promising avenue for enhancing patient outcomes.

## Introduction

Supracondylar humerus fractures are the most common type of elbow fractures in children, accounting for approximately 17.9% of all pediatric fractures (1). Neurological injuries are a common complication associated with these fractures, with reported incidence rates ranging from 11% to 15% (2–5). These nerve injuries related to fractures can further be categorized into traumatic and iatrogenic, with traumatic injuries comprising approximately 12%–20% (2, 5, 6) and iatrogenic injuries ranging from 2% to 6% (7, 8). Fractures commonly result in damage to the radial and anterior interosseous nerves (2, 9, 10), while iatrogenic causes more frequently affect the ulnar nerve (3, 11, 12).

Kirshner- wires (K-wires) fixation is a common treatment method for pediatric supracondylar humerus fractures. However, there is a 4% chance of injuring the ulnar nerve when using crossed K-wires technique (13). Currently, there is no optimal treatment for iatrogenic ulnar nerve injuries. Some experts suggest treating the fracture first and removing the K-wires after bone healing (14, 15); Others believe in early removal or repositioning of the K-wires (16), and/or early ulnar nerve exploration (7, 17). A systematic review indicates that if k-pins are removed at the scheduled time (approximately 3.5 weeks), with no premature extraction, approximately 91% of patients achieve full recovery, with an average recovery time of 4.5 months. Conversely, patients undergoing early k-wires removal or nerve exploration (around 3 days postoperatively) exhibit an 85% full recovery rate, with an average recovery time of 13.6 weeks (18). However, there is a lack of clear guidance on treatment strategies and timing for patients who show no improvement several months after k-wires removal.

Over the past decade, the supercharge end-to-side (SETS) anterior interosseous nerve (AIN) to ulnar motor nerve transfer (e.g., pronator quadratus muscle, PQ) has become increasingly popular as a means of enhancing intrinsic recovery in ulnar neuropathy (19). This transfer procedure is believed to offer faster and more thorough reinnervation of the intrinsic muscles compared to regeneration process solely from the proximal ulnar nerve (20). Current guidelines (21) indicate that there are three key considerations to determine whether the supercharge nerve transfer technique is suitable for a patient: the extent of ulnar axonal loss, the condition of the recipient intrinsic muscles (such as ongoing denervation), and the availability of a normal donor anterior interosseous nerve. The extent of ulnar axonal loss can be categorized into two types. The first type is a demyelinating abnormality (Sunderland first-degree injury or Seddon neurapraxia), where patients exhibit slowed conduction velocity across the elbow but normal compound muscle action potential amplitude (CMAPa) and typically recover with primary surgery at the elbow. The second type involves axonal loss (Sunderland second- or third-degree injury, or axonotmesis), where patients show reduced CMAPa and may benefit from a supercharge nerve transfer.

This study focuses on a 13-year-old child who suffered iatrogenic ulnar nerve injuries after cross K-wires fixation for supracondylar distal humerus fracture. Despite the removal of the K-wires 1.5 months later, significant intrinsic muscle atrophy and weakness persisted, with no neural recovery observed even after an additional four months of conservative treatment. The patient subsequently underwent neurolysis of ulnar nerve and supercharged end-to-side anterior interosseous nerve transfer, leading to a favorable outcome.

## Case presentation

### Present illness

**Figure 1:**
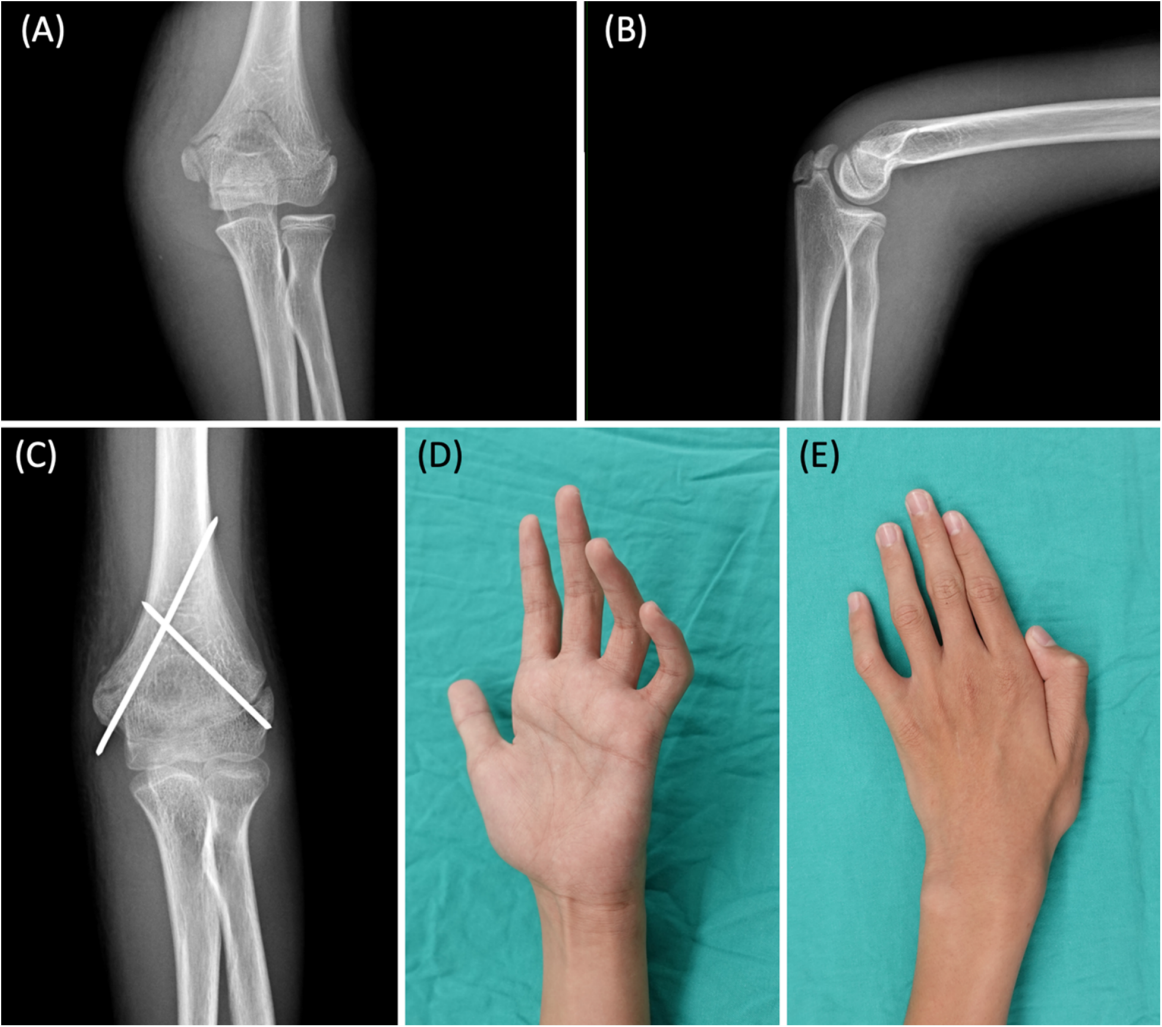
**(A)** AP view of the fracture before percutaneous fixation with K-wires. **(B)** Lateral view. The images show a humeral supracondylar linear fracture, which could have been treated conservatively, but the patient received K-wire fixation at another hospital. **(C)** Left distal humerus supracondylar fracture, status post closed reduction and internal fixation with cross k-wires fixation. **(D)** Marked claw deformity of the 4th and 5th fingers. **(E)** Intrinsic muscle atrophy with weakness in thumb and finger adduction (positive Froment sign and Wartenberg sign).

A 13-year-old boy with no known underlying disease, experienced left elbow pain following an accidental fall. Upon initial evaluation at other hospital, the x-ray revealed left distal humerus supracondylar linear fracture (Figures 1A,B), which could be treated nonoperatively with long arm cast. However, on the same day, he underwent closed reduction and cross k-wires fixation (Figure 1C). Symptoms and signs of ulnar neuropathy were observed immediately postoperatively and persisted even after the removal of the Kirschner wires 1.5 months post-fracture fixation. Furthermore, despite four additional months of conservative treatment involving Vitamin B supplementation and rehabilitation, there was no improvement in his clinical condition. Therefore, he was referred to our outpatient department (OPD) for further evaluation and treatment.

**Figure 2:**
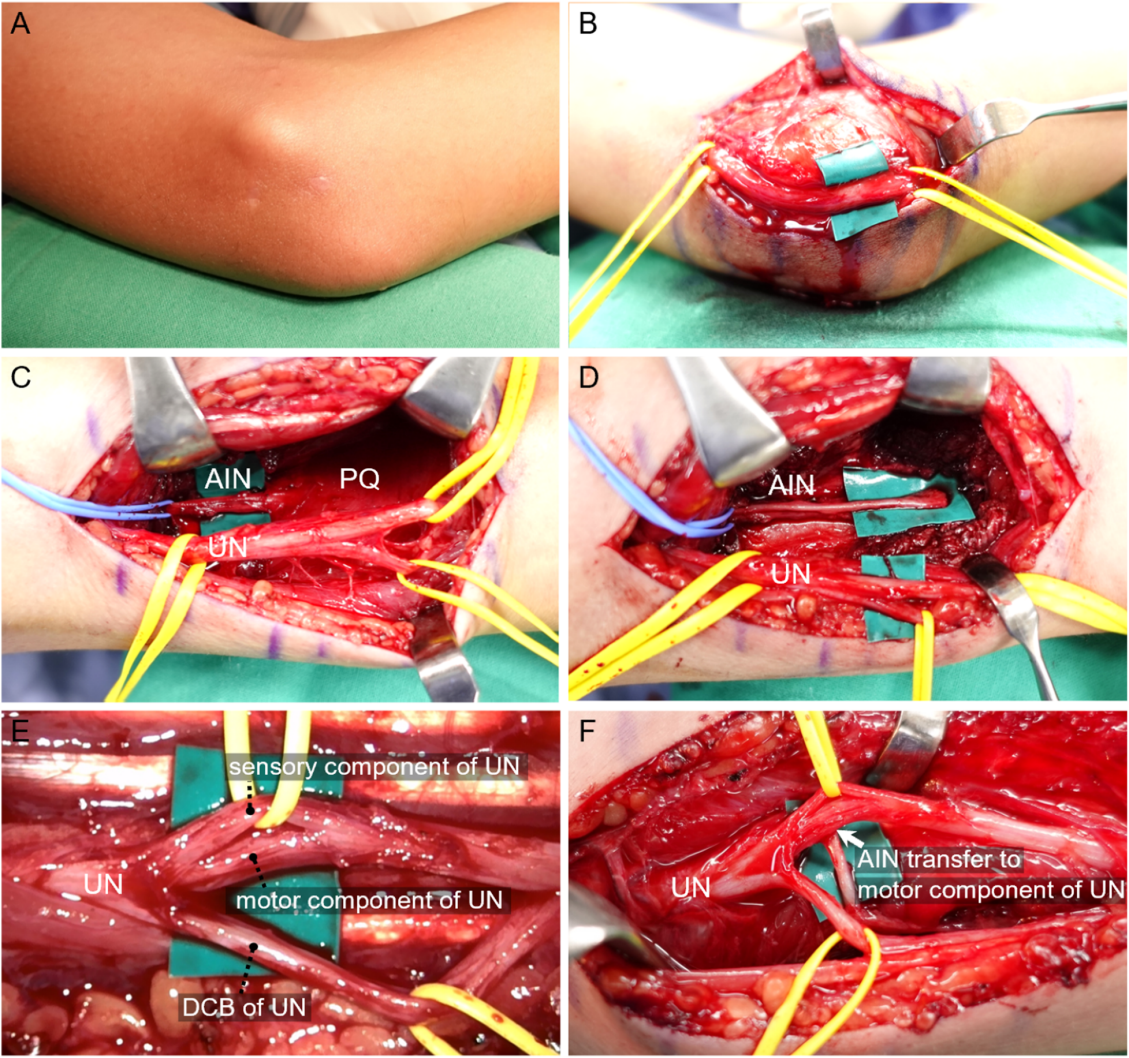
**(A,B, elbow)**. **(A)** Scars of previous k-wires fixation. **(B)** No notable adhesions, evident ruptures, or neuroma formations were found along the ulnar nerve pathway. However, slight scarring within nerve fascicles suspected to be caused by K-wire penetration was observed. (Above green patch) **(C–E, Forearm)**. **(C)** AIN and the PQ muscle were exposed. **(D)** AIN was traced to the AIN- PQ muscle junction first, and further dissected distally by releasing the PQ muscle. **(E)** The motor component of the ulnar nerve, located between sensory component and the dorsal cutaneous branch of ulnar nerve, was further dissected. **(F)** AIN was transferred to the dorsal surface of ulnar nerve motor component. (White arrow). AIN, Anterior interosseous nerve; PQ, pronator quadratus; UN, ulnar nerve; DCB, dorsal cutaneous branch.

During the physical examination at our OPD, the patient exhibited numbness in both the dorsal and volar regions of the hypothenar area, marked claw deformity of the 4th and 5th fingers (Figure 1D), intrinsic muscle atrophy with weakness in thumb and finger adduction (positive Froment sign and Wartenberg sign) (Figure 1E), and diminished power in 4th and 5th finger flexion. A penetrative scar was observed on the medial side of the elbow, located between the medial epicondyle and olecranon (Figure 2A). However, there was no evidence of skin tethering, and the Tinel sign was not elicited over the scar. The patient's grip strength, pinch strength, and thumb adduction strength were measured at 7 kg, 5 kg, and 1 kg, respectively. Additionally, the Quick Disability of the Arm, Shoulder, and Hand (Quick DASH) score was 37.5. EMG/NCV performed 3 months after fracture surgery also indicated severe left ulnar neuropathy at the elbow level, characterized by active denervation. After thorough preoperative discussions, the patient and his family decided to proceed with surgical interventions to improve his function.

### Operation

The surgical procedure was executed in three sequential steps: cubital tunnel release, nerve transfer and tendon transfer. Initially, a curved incision was made over the medial aspect of the elbow to fully release the cubital tunnel and assess the condition of the ulnar nerve. Upon examination, no notable adhesions, evident ruptures, or neuroma formations were found along the ulnar nerve pathway. However, slight scarring within nerve fascicles suspected to be caused by K-wire penetration was observed (Figure 2B). Motor responses were confirmed to be absent below the lesion site through the use of electric nerve stimulation. Subsequently, through a longitudinal incision made along the Flexor Carpi Ulnaris, extending 6–12 cm proximal to the wrist joint, the underlying ulnar nerve and dorsal cutaneous branch of the ulnar nerve were identified. The tendons of the Flexor Digitorum Profundus(FDP) and Flexor Digitorum Superficialis were retracted radially to expose AIN and the PQ muscle, which were located beneath these structures (Figure 2C). After confirming that the PQ muscle response was robust following electric stimulation of the AIN, the AIN was traced to the AIN-PQ muscle junction. It was then further dissected distally by releasing the PQ muscle, thereby gaining additional nerve length (Figure 2D). Under the microscope, the motor component of the ulnar nerve, located between sensory component and the dorsal cutaneous branch of ulnar nerve, was further dissected and prepared to serve as the recipient nerve (Figure 2E). Epineurial and perineurial windows measuring 2 mm were created and then the anterior interosseous nerve (AIN) was transferred to the dorsal surface of the ulnar nerve motor component (Figure 2F). This nerve transfer was accomplished using an end-to-side method, employing 10-0 Nylon sutures under microscope after checking no tension, kinking or impingement during range of motion.

Finally, a tenodesis of the FDP of the 4th/5th fingers to the FDP of the 3rd finger was performed using 1-0 Vicryl, with the fingers positioned in a slightly flexed posture.

### Post-operative course

**Figure 3:**
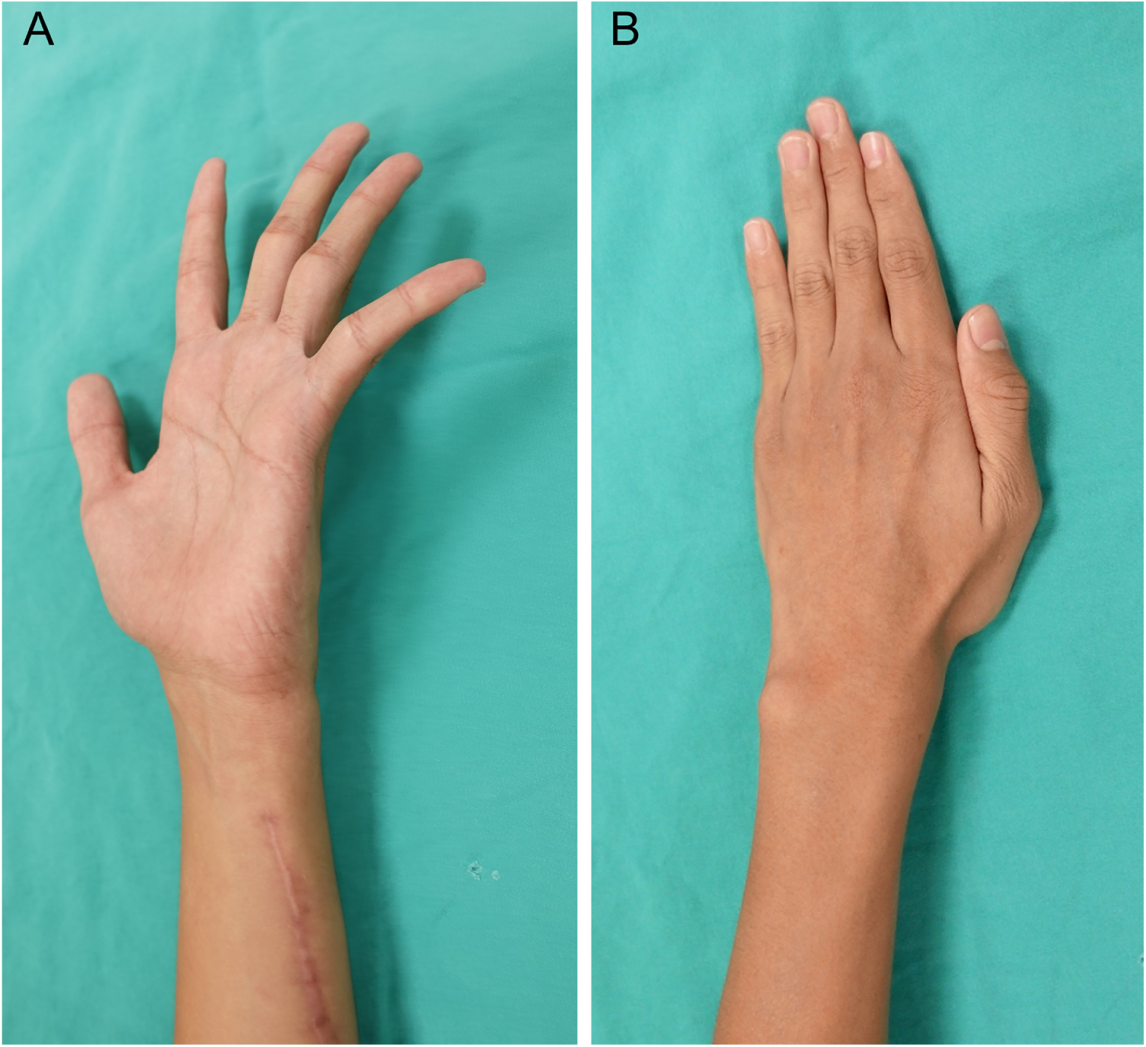
**(A)** After a six-month OPD follow-up, the 4th/5th finger claw deformity returned to normal. **(B)** No atrophy of intrinsic muscle or weakness in thumb and finger adduction.

The rehabilitation protocol was as follows: finger flexion/extension exercises begin one week postoperatively, forearm pronation/supination and finger abduction/adduction exercises start two weeks later, combining forearm pronation/finger adduction with resistance commence after one month, and full activity is allowed after two months. After a six-month OPD follow-up, the 4th/5th finger claw deformity returned to normal (Figure 3A) without intrinsic muscle atrophy (Figure 3B). Grip strength increased from 7 kg to 22 kg, while pinch strength remained at 5 kg. Thumb adduction strength improved from 1 kg to 3.5 kg. The Quick DASH score improved from 37.5 to 0. No acute or chronic complications were recorded postoperatively. The preoperative and postoperative parameters regarding function and strength measurements were summarized in Table 1.

**Table 1:** The preoperative and postoperative function and strength parameters.

	Pre-OP	Post OP 6M
Quick DASH score	37.5	0
Grip (kg)	7	22
Pinch (kg)	5	5
Thumb adduction (kg)	1	3.5

DASH, disability of the arm, shoulder, and hand.

## Discussion

Pediatric ulnar nerve injuries are common, yet there is currently no consensus on the optimal treatment for such injuries in children. Previous study suggests that the timing of K-wire removal, whether early or at the scheduled time, appears to have minimal impact on prognosis (18). Therefore, waiting for approximately one month for fracture healing before removing the K-wire seems to be a reasonable approach. We followed this protocol, extracting the K-wire approximately 1.5 months after fracture fixation. However, 4 months after k-wire removal, the nerve not only failed to recover completely but also exhibited signs of muscle atrophy. This contrasts with the literature, which reports a 90% average complete nerve recovery within 3 months (18).

There is currently no consensus on the timing of surgical intervention (22). According to Dahlin et al., if neuropathy persists for more than one year, permanent muscle atrophy may occur, potentially leading to irreversible functional loss. It is advisable to monitor these types of injuries closely for a period of 6 months, and potentially up to a year, before considering surgical exploration (23, 24). Since we lack pediatric literature on this topic, we have referred to adult experiences. The nerve junction site of the AIN supercharged end-to-side nerve transfer is closer to the intrinsic muscles, which may shorten the nerve regeneration time to target muscles. In adult studies, the average time to recovery was 7 months (25). Considering that this patient had already exhibited signs of muscle atrophy, we opted to perform the surgery six months after the onset of symptoms. This timing would allow adequate time for nerve regeneration, aiming to prevent irreversible muscle atrophy.

Regarding conservative treatment failure in pediatric ulnar neuropathy patients, there is a lack of discussion on surgical options in existing literature. A recent randomized control trial regarding ulnar nerve decompression and transposition with vs. without SETS motor nerve transfer in adult patient with advanced cubital tunnel syndrome (Mean age 56 and 54 in years), showed that SETS motor nerve transfer demonstrated significant superior of postoperative pinch strength, CMAPa of the first dorsal interossei and abductor digiti minimi. Additionally, in the SETS group, 67% of patients achieved good to excellent results compared to only 35% in the control group (26). In adult patients, SETS indeed enhances the effectiveness of surgery.

It is worth noting that, currently, most SETS nerve transfers of the terminal AIN to the PQ muscle in adults follow the approach pioneered by Dr. Mackinnon (19). However, unlike Mackinnon's technique, we adjusted the nerve coaptation site to the dorsal side to minimize the distance of the transfer route and reduce nerve tension.

Additionally, we employed a shorter longitudinal incision in the forearm and opted not to release the Guyon canal, owing to the absence of wrist trauma and low risk of associated compression neuropathy at the Guyon canal. In the distal forearm, the motor component of the ulnar nerve is consistently located between the sensory component and the dorsal cutaneous branch of the ulnar nerve. Consequently, there is no necessity to trace it retrogradely from the ulnar nerve's bifurcation in the Guyon canal.

Current concept suggests faster nerve recovery in children due to the superior capacity of children's central nervous system to adapt to external or internal environmental changes (neural plasticity) and the shorter recovery distance from the axon repair site to the target muscle (27). However, there is no specific literature addressing the optimal surgical approach for pediatric ulnar nerve neuropathy when conservative treatment fails. This paper is the first to propose the use of neurolysis and supercharge for pediatric ulnar neuropathy. The simultaneous use of both techniques is based on evidence indicating that increasing supercharge can indeed enhance postoperative functional outcome and intrinsic muscle strength.

For this case report, our advantages include the patient's excellent recovery after the nerve transfer surgery and the fact that there are currently few studies discussing the treatment of ulnar nerve neuropathy in children, with most focusing on adults. Moreover, there is no existing literature addressing the prognosis of using the supercharge technique in children, so we believe this article can offer a new perspective. However, the limitations include the aesthetic issue of a scar over the volar forearm area, which the patient and his family found acceptable. Additionally, the patient may lose some function of the PQ muscle, although this loss is not noticeable. Lastly, as a case report, it involves only a single patient, the sample size is too small, and there is no control group for comparison.

## Conclusion

In patients with ulnar nerve neuropathy resulting from cross pinning after pediatric supracondylar humerus fracture, performing neurolysis with supercharged end-to-side anterior interosseous nerve to ulnar motor nerve transfer yields favorable outcomes when there is no complete neural recovery after K-wire removal and subsequent four months of conservative treatment.

## Data Availability

The original contributions presented in the study are included in the article/Supplementary Material, further inquiries can be directed to the corresponding author.
